# Colorimetric Aptasensor of Vitamin D3: A Novel Approach to Eliminate Residual Adhesion between Aptamers and Gold Nanoparticles

**DOI:** 10.1038/s41598-018-31221-y

**Published:** 2018-08-28

**Authors:** Omar A. Alsager, Khalid M. Alotaibi, Abdullah M. Alswieleh, Baraa J. Alyamani

**Affiliations:** 1 National Center for Irradiation Technology, Nuclear Science Research Institute, King Abdulaziz City for Science and Technology, P.O. Box 6086, Riyadh, 11442 Saudi Arabia; 2 Department of Chemistry, College of Science, King Saud University, P.O. Box 2455, Riyadh, 11451 Saudi Arabia

## Abstract

Colorimetric aptasensors based on gold nanoparticles (AuNPs) commonly feature ssDNA probes nonspecifically adsorbed to surface gold particles. A major limitation of this versatile method is the incomplete dissociation of the adsorbed nontarget binding segments of the aptamer sequence upon target binding. This results in weak or nonexistent sensor performance by preventing the particles from aggregating when the optimized salt concentration is added. Rather than removing the nonbinding nucleotides flanking the binding region of the aptamer, proposed herein is an alternative strategy, simply introducing a centrifugation and resuspension step after target recognition that eliminates residual binding between the aptamer and the surface of the particles. The performance of two different vitamin D3 (VTD3) aptamers were tested. The method enhanced the performance of the sensor that used the higher detection limit (1 µM) aptamer by fourfold. The superiority of the proposed method became apparent in a nonworking colorimetric sensor became a highly sensitive sensor with a one nanomolar detection level and excellent discrimination against potential interfering molecules including VTD2 when the centrifugation and resuspension process was implemented. The level of VTD3 in human blood was determined colorimetrically after extraction with n-hexane. The results were in agreement with those obtained by HPLC. The proposed method could be applied to aptamers targeting small molecules with no need to reprocess the SELEX-isolated sequence by knowing the binding region and removing the flanking primers.

## Introduction

Gold nanoparticles (AuNPs) possess attractive physical and chemical properties, such as large surface area, chemical enhanced reactivity, and localized surface plasmon resonance (LSPR)^1–3^. LSPR is the most remarkable property of AuNPs that provides colloidal suspensions with orders of magnitude greater extinction coefficients than conventional dyes^4^. Clearly distinguishable colors in the visible spectrum can be seen when the particles are well dispersed in comparison with when they are aggregated. Thus, appropriate design of the chemical interaction between an analyte and the particle surroundings will lead to a change in color (red to blue or vice versa), allowing the visual detection of the target analyte^5,6^. As a consequence, the detection of molecules with high significance in the medical, clinical, food safety and environmental fields has been reported recently using AuNPs with the advantage of various recognition elements and sensing formats^3,5,7^. Targets include DNA^8^, proteins^9,10^, a wide range of organic molecules^11–14^, and inorganic metal ions^15,16^.

Over the last decade, ssDNA aptamers have emerged as promising alternatives to antibodies and have been used as a biological recognition element for biosensing, disease diagnosis, and therapeutic application^17–21^. They are generated by an *in vitro* combinatorial chemical process called systematic evolution of ligands by exponential enrichment (SELEX) to specifically and selectively bind targets^22–24^. Aptamers have advantages over monoclonal antibodies in that they are greatly specific, stable, relatively small biomolecules, more efficient in binding small targets (such as steroidal hormones, pesticides, and antibiotics), chemically synthesized in bulk quantities, and nonimmunogenic.

**Figure 1:**
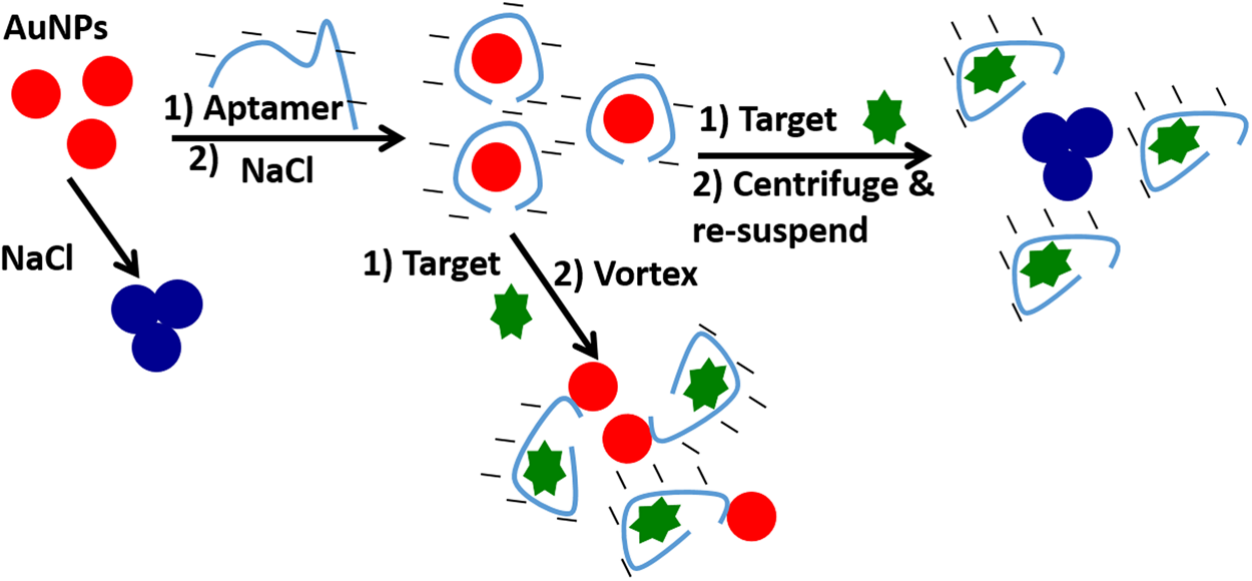
AuNP colorimetric aggregation-based sensing method. The Figure illustrates the proposed suppression effect of nonbinding flanking nucleotide on target binding signals, adhering to the particles after target detection, which prevents aggregation, and the role of the proposed method (centrifugation and resuspension) to eliminate the residual adhesion of these nonbinding sequences.

Among various signal transduction schemes previously incorporated in aptamer-based sensors, such as electrochemical^25^, size-based^26^, fluorescence^27^, and lateral flow^28,29^, those exploiting aptamer nonspecific adsorption and AuNP aggregation are emerging as one of the most effective and easily implemented methods^13,14,30–35^. As shown in Fig. 1, the negatively charged aptamer sequences are nonspecifically adsorbed on the surface of citrate-capped AuNPs via the nitrogen bases of the DNA, resulting in well-dispersed negatively charged AuNP-aptamer conjugates in moderately high ionic strength medium. However, the target induced conformational change within the aptamer leads to desorption (in most cases^36^) of the aptamer from the surface by reducing the aptamer affinity to the particles. Subsequently, AuNPs aggregate in response to the presence of target concentration by turning the colloidal gold solution from red to purple–blue^6,34^.

This colorimetric sensing method operates based on driving the system between two competitive interaction dynamics: A) affinity between the aptamer sequence and the particles, and B) affinity between the aptamer and the target. The interaction between the aptamer and its target is highly specific, and only a small fragment of the aptamer is responsible for small molecular weight target binding (located in the core random region). However, aptamer-particle interaction is nonspecific, and presumably all the aptamer bases facing the particle surface (not part of the folded structure) could be involved if structurally available. If the aptamer-particle binding is too strong, the sensitivity of target recognition will be diminished or could cause sensor failure^30,37^. This issue was partially solved by removing excess flanking nucleotides not directly committed to the target binding of the parent aptamer sequence to minimize the interaction with the particles and to enhance the sensor sensitivity. The approach resulted in reasonable enhancements in the performance of various colorimetric sensors including the case of detecting tetracycline (500-fold performance-enhancement for 8-mer vs. 76-mer)^31^, 17β-estradiol (25-fold lower detection limit for 35-mer vs. 75-mer)^34^, and acetamiprid (3.3-fold performance-enhancement for 37-mer vs. 49-mer)^35^.

However, the process is time consuming, requires extensive characterization of many aptamer versions generated from the original parent aptamer, depends on a trial and error approach and could adversely impact the affinity, specificity, and stability of the aptamer by removing bases involved in the target binding. Additionally, the approach is impractical when using the colorimetric sensing scheme to evaluate tens of aptamer sequences generated from SELEX process for sensitivity^38^.

In this study, an alternative strategy to removing residual affinity between AuNPs and excess nontarget binding nucleotides is proposed. We hypothesized that the residual attachment between AuNPs and nucleotides not directly committed to target recognition could be avoided after target recognition (when the affinity between the aptamer and particles is reduced by target-recognition^6^) by applying a centrifugation and resuspension step, routinely used to concentrate and purify AuNPs from access ligands as well as to transfer the particles from one medium to another^33,39^. Upon target recognition, the aptamer undergoes a conformational change that favors the target molecule over AuNPs, which is the basis of many colorimetric sensors^30,37^. The centrifugation and resuspension approach could apply a pressure to force a complete dissociation of aptamer sequences that are still adhered to the surface of the particles after target recognition. The approach was applied to develop a colorimetric sensor for Vitamin D3 in buffer and extracted from blood samples. Two previously isolated aptamers sequences were used, the Bruno 69-mer aptamer^40^ and the 56-mer Lee aptamer^37^.

VTD3 is a vital biological component in the human body. It is known for its basic role in many biological functions such as mineralization of teeth and bones through regulation of calcium and phosphorus homeostasis^41,42^. There is emerging evidence of the role of VTD3 in protecting against the risks of malignant neoplasms, cardiovascular disease, diabetes, osteoporosis, and other bone disorders^41,43,44^. The standard quantitative measuring protocols of VTD3 levels are high-performance liquid chromatography (HPLC) and enzyme-linked immunosorbent assay (ELISA)^45,46^. These methods are available only in specialized laboratories^47^, and ELISA is unstable under harsh conditions, and requires a sophisticated and high production cost approach^48^. Therefore, there is an urgent need for rapid and sensitive methods for the detection of VTD3 in point-of-care diagnostics applications.

We have successfully applied the proposed method (Fig. 1) to improve the sensor performance by 4-fold when using Bruno aptamer, i.e., greater degree of aggregation response. The practicality of our proposed method became apparent when it turned a nonoperating colorimetric sensor (using the Lee aptamer) into a sensor with a one nanomolar level of detection, excellent discrimination against potential interfering molecules, including VTD2, and ability to determine the level of VTD3 in blood after a rapid and simple n-hexane extraction step. Our work provides a highly effective sensor scheme for the detection of 1 nM VTD3, as well as a simple methodology that is broadly applicable to the growing number of colorimetric aptasensors. More generally, this work demonstrates the benefit of introducing a simple centrifugation step and its positive effect on this particular colorimetric signal transduction platform.

## Results and Discussion

### Colorimetric sensors construction and characterization

The utilization of the plasmonic response of AuNP-aptamer conjugates to design a reactive sensor for the quantification of the small molecular target VTD3 was initially demonstrated using the conventional sensing format^30,34,49^ shown in Fig. 1. An important consideration in such a system is that the affinity of the free aptamer in solution could be affected by adsorption on the AuNP surface. Aptamer sequences adhered to the AuNP surface via multiple nucleotides, which could alter the thermodynamic and kinetic properties of target binding. Therefore, we began to study the aptamer adsorption and target induced-desorption of VTD3 aptamers by conducting salt-induced aggregation. Additionally, surface potential measurements were conducted to examine how different states (adsorbed and desorbed) of the aptamer will affect the AuNP surface properties.

**Figure 2:**
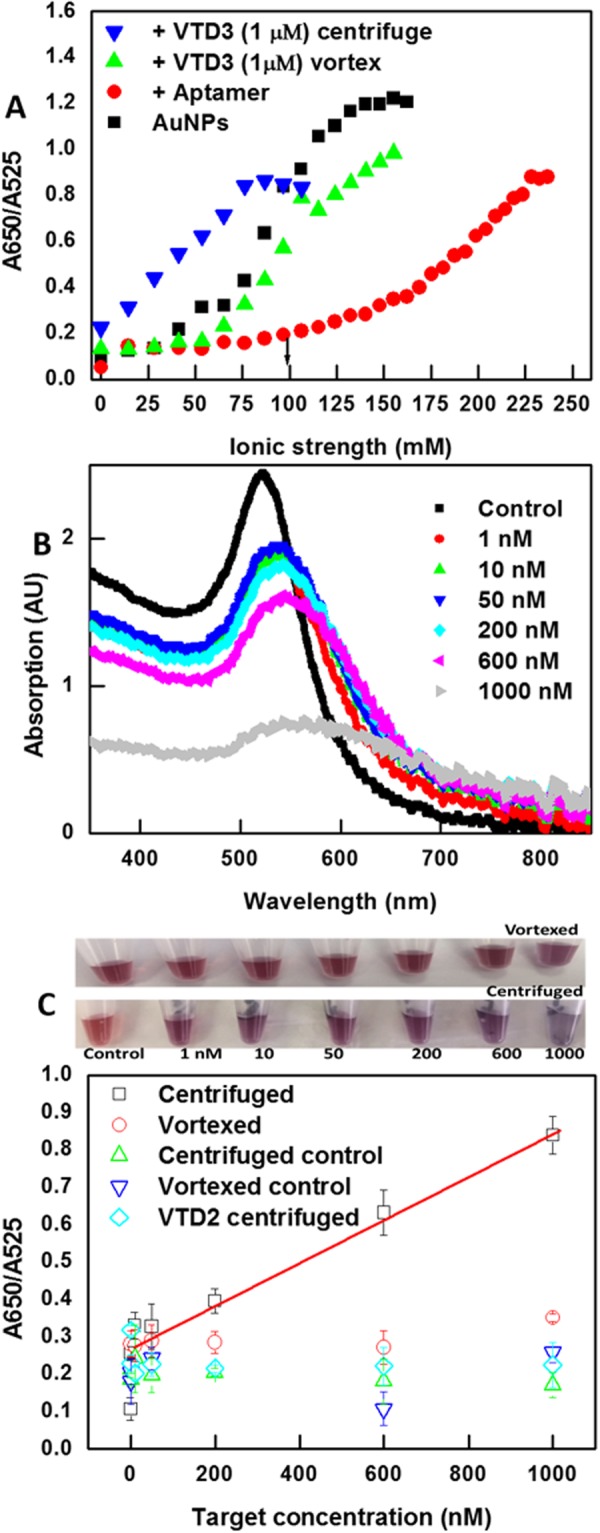
(**A)** Salt tolerance experiments with bare AuNPs, AuNP-Lee aptamer (100 nM), and AuNP-Lee aptamer + VTD3 (1 µM) when using vortexing and centrifugation sensing methods. The optimal salt concentration is indicated by the black arrow. (**B)** UV-visible spectra of the sensor performance after independent incubation with control (buffer only) and increasing VTD3 concentrations and application of centrifugation and resuspension step. (**C)** Colorimetric aptasensor response towards a range of VTD3 concentrations using the AuNP-Lee aptamer (vortexed and centrifuged) compared with control experiments using AuNP-70-mer random ssDNA exposed to the same experimental steps. Top panels show photos of the sensor response in both cases, i.e., vortexed and centrifuged conditions. The sensor response to a range of VTD2 concentrations is also shown. Error bars indicate standard deviation of the mean of three independent experiments starting from particle functionalization.

To develop a sensitive AuNP-based colorimetric aptasensor, the minimum aptamer concentration used to stabilize AuNPs should be determined to prevent the presence of solution-free aptamers that will not contribute to the colorimetric sensing and therefore will diminish the sensitivity^34,49^. As shown in Fig. 2A, the adsorption of 0.3 nmoles of Lee aptamer (which yields a 100 nM aptamer concentration and an aptamer/particle ratio of 9/1) resulted in a remarkable resistance against salt-induced aggregation compared to bare AuNPs. This is anticipated from the degree of aggregation: the ratio of AuNP absorption at 650 nm (blue, aggregated AuNPs) and 525 nm (red, well-dispersed AuNPs). It should be noted that the lower aptamer concentration resulted in an irreproducible particle protection, while the higher concentration reduced the sensor sensitivity towards the target, which is consistent with previous studies^34,49^. The enhanced tolerance towards salt aggregation (Fig. 2A) is evidence of the successful adsorption of the negatively charged polyionic aptamer, which is also clearly indicated by the increased surface potential values from PLS measurements (Fig. S2B: −23 mV vs. −42 mV for bare AuNPs and AuNP-Lee aptamer, respectively).

**Figure 3:**
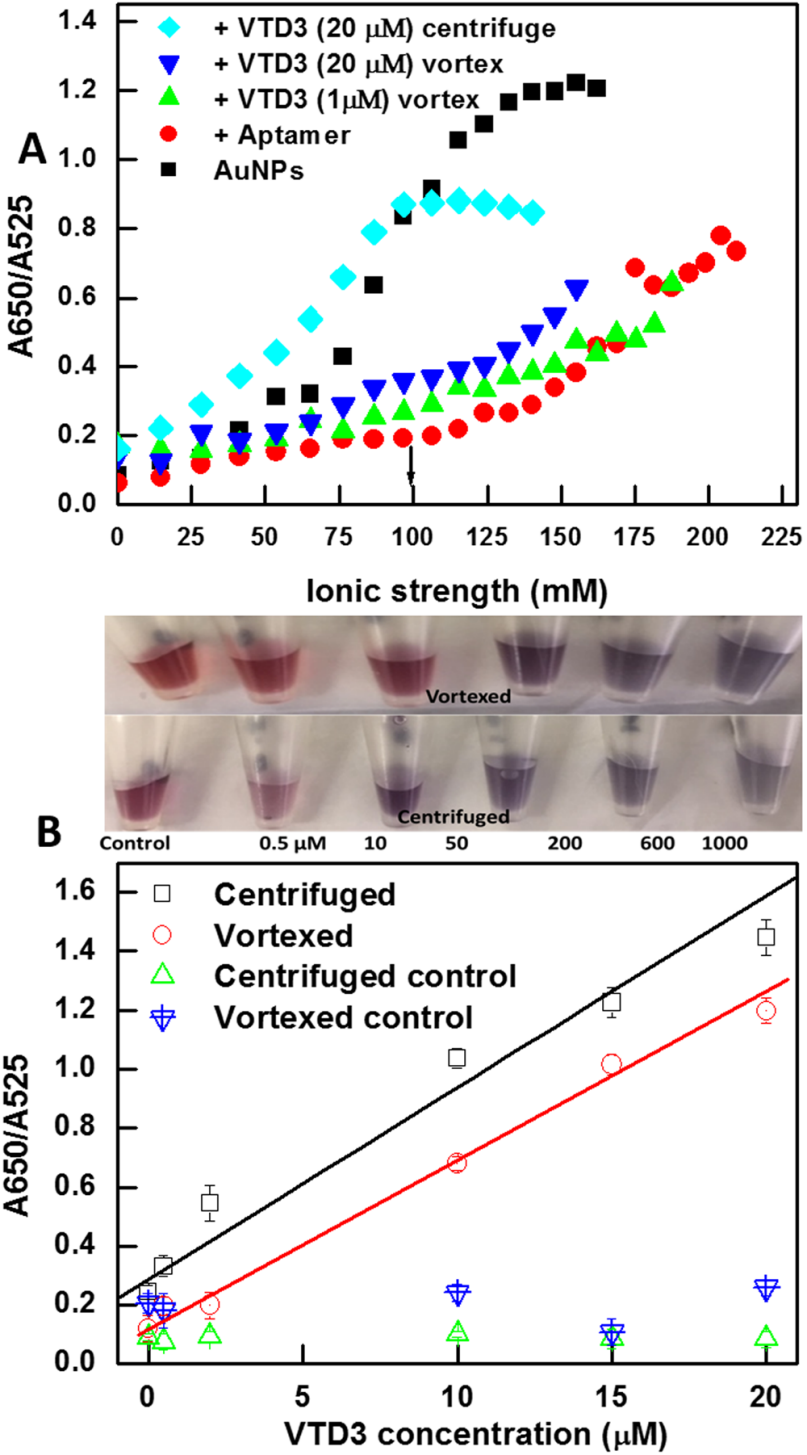
(**A)** Salt tolerance experiments of bare AuNPs, AuNP-Bruno aptamer (100 nM), and AuNP-Bruno aptamer + VTD3 (1 µM and 20 µM, respectively) when using vortexing and centrifugation sensing methods. Optimal salt concentration is indicated by the black arrow. (**B)** Colorimetric aptasensor response towards a range of VTD3 concentrations (µM range) using the AuNP−Bruno aptamer (vortexed and centrifuged) compared with control experiments using AuNP-70-mer random ssDNA exposed to the same experimental steps. Top panels show photos of the sensor response in vortexed and centrifuged conditions for both cases. Raw UV-visible spectra are provided in the Supporting Information. Error bars indicate standard deviation of the mean of three independent experiments starting from particle functionalization.

It is expected that different aptamers will have different affinities for the AuNP surface due to the differences in the structures, compositions, and lengths^33,34^. For example, the affinities of different DNA bases to AuNPs decreased in the order C > G > A > T when probed by isothermal titration calorimetry studies^50^. On the other hand, thermal desorption measurements found that the affinities to gold surfaces decreased in the order G > C ~ A > T^51^. FTIR spectroscopy^52^, and AuNP aggregation studies^53^, agree that oligo (dT) has the weakest affinity to gold surfaces and both measurements arrived at a different order of affinities; A > C ≥ G > T; than the previous methods. Additionally, longer DNA sequences appeared to provide greater degree of protection against the aggregation induced by salt addition^34^. It is therefore essential to understand experimentally how each ssDNA composition will affect the adsorption on AuNPs and subsequent sensing parameters under the experimental conditions conducted in present study. The abovementioned characterization (with lee aptamer) was repeated with AuNP-Bruno aptamer, shown in Fig. 3A. Similarly, adsorbing Bruno aptamer on AuNP surface at a ratio of 9/1 resulted in an increased resistance towards salt-induced aggregation, evidenced from the behavior monitored by the absorption ratio at 650 nm/525 nm. Surface potential measurements conducted by PLS indicated that the aptamer adsorption increased the surface potential from −23 mV to −68 mV. The higher surface potential value for Bruno aptamer compared to Lee aptamer (−68 mV vs. −42 mV) could be attributed to the extra 13 bases contained in Bruno aptamer as well as its likely stronger affinity to the AuNP surface due to the more abundant unfolded nucleotides within the aptamer structure (refer to mfold secondary structure shown in Fig. S2).

Incubation of aptamer systems with VTD3 (1 µM) followed by vortexing led to the loss of salt resistance, confirming that VTD3 caused the aptamer to detach from the surface, at least partially (Figs 2A and 3A). Note that the Bruno aptamer responded weakly to 1 µM VTD3, and even 20 µM VTD3 does not reach the level of dissociation observed with Lee aptamer incubated with 1 µM of VTD3 (closely approaching the signal of bare AuNPs). This is consistent with the previously mentioned adsorption data from PLS (Fig. S3) and our secondary structure analysis (Fig. S2), as the Bruno aptamer appears to adsorb more strongly than the Lee aptamer. We made sure that our observation in the salt titration experiments arose from specific aptamer-target interaction by repeating the experiments with a random ssDNA (Fig. S12). Additionally, Fig. S3 shows that incubating the Lee and Bruno aptamer systems with the target at saturation level reduced the surface potential values from −42 mV to −34 mV for the Lee aptamer and from −68 mV to −44 mV for the Bruno aptamer, indicating that target-aptamer complex formation and partial surface dissociation. Conducting the same surface potential experiments with the random ssDNA showed no alternation in surface potential values (Fig. S3) which confirms that our observation arose from specific aptamer-target interaction.

### Performance of the VTD3 colorimetric sensor with the vortexing approach

Having confirmed that both aptamer sequences adsorbed on the AuNP surface and the interaction with VTD3 promoted the desorption of the sequences, we proceeded to apply the aptasensor using the conventional protocol implemented in previous colorimetric aggregation studies (gentle shaking or vortexing)^13,14,30–35^. The salt concentration was optimized prior to target sensing whereby the AuNP-aptamer suspension is brought to the edge of stability (100 mM NaCl indicated by the black arrow in Fig. 2A) and the introduction of VTD3 triggers significant aggregation. As seen in Fig. 2C, the only significantly resolvable sensor signal was associated with 1 µM VTD3 concentration (labeled as vortexed). The sensor response was verified by replacing the specific VTD3 Lee aptamer with a random 70-mer ssDNA and confirming that the signals arose from a specific VTD3-aptamer interaction. The exposure of organic molecules to AuNPs was previously found to cause various degrees of aggregation^54^. Thus, the interaction of the particles with increasing VTD3 concentration (1 nM–1000 nM) was examined, as shown in Fig. S13. It was concluded that VTD3 does not cause particle aggregation in the examined nanomolar concentrations. The lowest colorimetrically detectable target concentration is 1 µM (top panel of Fig. 2C), which is consistent with the detection limit reported by Lee *et al*. for the same aptamer and using the same detection format^37^. Thus, the performance of the Lee aptamer under the vortexing conditions does not construct a senor with a sensitivity behavior.

Performing the colorimetric sensor assay with the Bruno aptamer under the same experimental conditions as those with the Lee aptamer and using the optimized salt concentration (100 mM indicated in Fig. 3A) resulted in a sensitive response towards VTD3 at concentrations in the µM range. The lowest detected concentration of VTD3 is 0.5 µM (spectroscopically) with an excellent linearity (R^2^ = 0.99) and an analytical window covering the lower range of µM concentration. The lowest colorimetrically detected VTD3 concentration is 10 µM.

### Performance of the VTD3 colorimetric sensor with the centrifugation and re-suspension approach

An insufficient level of total VTD3 in human blood has been defined as less than 50 nM^55,56^, while sufficient levels were set as being between 70 nM and 130 nM^43,55^. VTD3 concentrations that exceed 240 nM are considered to be toxic^56,57^. Thus, even the successfully developed colorimetric aptasensor with Bruno aptamer cannot be utilized in point-of-care diagnostic applications due to its high detection limit, which exceeds the needed sensitivity by at least 10-fold. The failure in sensor performance with Lee aptamer and diminished sensitivity with Bruno aptamer could be due to their long sequences (69-mer and 56-mer) resulting in strong adhesion to the surface. This is evidenced from the fact that salt titration curves (Figs 2A and 3A) did not return to those of the bare AuNPs after treatment with VTD3 at saturation levels. Additionally, the surface potential values presented in Fig. S3E show that incubating VTD3 at saturation levels with the AuNP-aptamer systems did not return to the original values of bare particles after vortexing-assisted detection. Residual aptamer molecules remain adsorbed to the surface under these conditions, conferring electrostatic stability and thus diminishing their potential sensitivity.

This problem has been identified previously with a number of colorimetric aggregation sensors and solved by shortening the aptamer sequences by removing the excess nucleotides flanking the binding region of the aptamer. The approach resulted in reasonable enhancements in the performance of various colorimetric sensors including the case of detecting tetracycline (500-fold performance-enhancement for 8-mer vs. 76-mer)^31^, 17β-estradiol (25-fold lower detection limit for 35-mer vs. 75-mer)^34^, and acetamiprid (3.3-fold performance-enhancement for 37-mer vs. 49-mer)^35^.

However, utilizing this approach means that many aptamer versions generated from the original parent aptamer must be characterized. Additionally, the method depends on a trial and error approach, which could adversely impact the affinity, specificity, and stability of the aptamer by removing bases involved in the target binding. Eliminating the nonbinding nucleotides becomes impractical when utilizing the colorimetric sensing scheme to evaluate a vast number of aptamer sequences generated from the SELEX process for sensitivity^38^.

Figure 2C shows that the issue could be solved by simply introducing a centrifugation and resuspension step, as illustrated in Fig. 1. After target incubation and salt addition, centrifuging and resuspending the AuNP conjugates (in the same supernatant) remarkably turned the Lee aptamer sensor into a highly sensitive recognition tool for VTD3 with a detection limit of 1 nM (defined as S/N > 3) and a good linear response (R^2^ = 0.97) covering the entire range of nM concentrations. Figure 2B presents the UV-visible spectra of the sensor response to the range of VTD3 concentration, which shows clear formation of aggregates as the VTD3 concentration increases. The 1 nM detection level of VTD3 can easily be seen by the naked eye (top panel of Fig. 2C). Incubation of VTD3 with a random 70-mer ssDNA-coated AuNPs did not show sensing signals when applying the same experimental steps. The sensor is highly reproducible; the error bars indicate standard deviation of the mean of three independent experiments starting from particle functionalization (Fig. 2C).

Figure 3B shows the generality of the centrifugation and resuspension process when improving the performance of the colorimetric sensor constructed with Bruno aptamer by 4-fold while maintaining the sensing characteristics identified earlier, except an enhancement in the colorimetric detection limit to reach 0.5 µM instead of 10 µM. Again, we ensured that the signals arose from specific aptamer-VTD3 interactions by replacing the aptamer with the random 70-mer ssDNA. The aggregate formation after target recognition was further examined by DLS for both sensor systems (Fig. S3A and C).

We further analyzed the effect of the centrifugation and resuspension approach on the apparent dissociation constants (Kd, app) of lee aptamer and Bruno aptamer. Langmuir model was previously implemented to obtain the Kd values of various aptamers binding to small molecules when the same type of colorimetric aggregation sensor was used^58^. Therefore, it was used in our case to analyze the developed approach and its effect on the Kd, apps (detailed in the Supporting Information, Fig. S14). It was found that there was no resolvable binding between lee aptamer and VTD3 when implementing the vortexing method. However, a Kd, app value of 220 nM was calculated from the non-linear fit of the data representing the centrifugation and resuspension approach, Fig. S14A. Similarly, a better Kd, app was calculated for Bruno aptamer when the centrifugation and resuspension was used compared to the vortexed based sensing (5 µM vs. 9.2 µM respectively), Fig. S14B. These results are consistent with our previously noted observation with the overall sensor performance and sensor sensitivity. The centrifugation and resuspension approach seems to eliminate the residual adhesion of non-binding aptamer segments to AuNPs, which makes the sensor more sensitive to the VTD3 concentrations.

A further investigation of the desirable effect of centrifugation and resuspension on the removal of residual adhesion of aptamer sequences was conducted by surface potential measurements. Fig. S3 shows that incubating the AuNP-aptamer with the target at saturation conditions followed by centrifugation and suspension retained the original surface potential value of bare particles, indicating that the centrifugation plays a fundamental role in removing residual sequences adhered to AuNP surface after the detection of VTD3. We verified our conclusions by repeating the same experiments with the random ssDNA (−46 mV) or AuNP-aptamer systems (−68 mV and −42 mV) with no target incubation and observed no alteration in the surface potential values. The surface potential results are consistent with the salt titration experiments presented in Figs 2A and 3A. The centrifugation and resuspension protocol eliminated the residual resistance of AuNP-aptamer-VTD3 conjugates observed with the vortexing method. Implementing the proposed method facilitates the complete removal of aptamer sequences from the surface, allowing the AuNP-aptamer-VTD3 populations to show bare AuNP-like behavior against salt titration. Repeating the salt titration experiments with the random ssDNA and implementing the centrifugation and resuspension protocol (Fig. S12) confirmed that our observation is due to specific aptamer-target interaction.

It should be noted that the optimized combination of centrifugation time and speed were found to be 13000 rpm for 5 min (Fig. S10**)**. Shorter time or lower speed resulted in incomplete separation of the particles from the supernatant. However, longer time promoted irreversible AuNP aggregation. Thus, the centrifugation force must be finely tuned.

### Selectivity of the developed VTD3 colorimetric sensors

**Figure 4:**
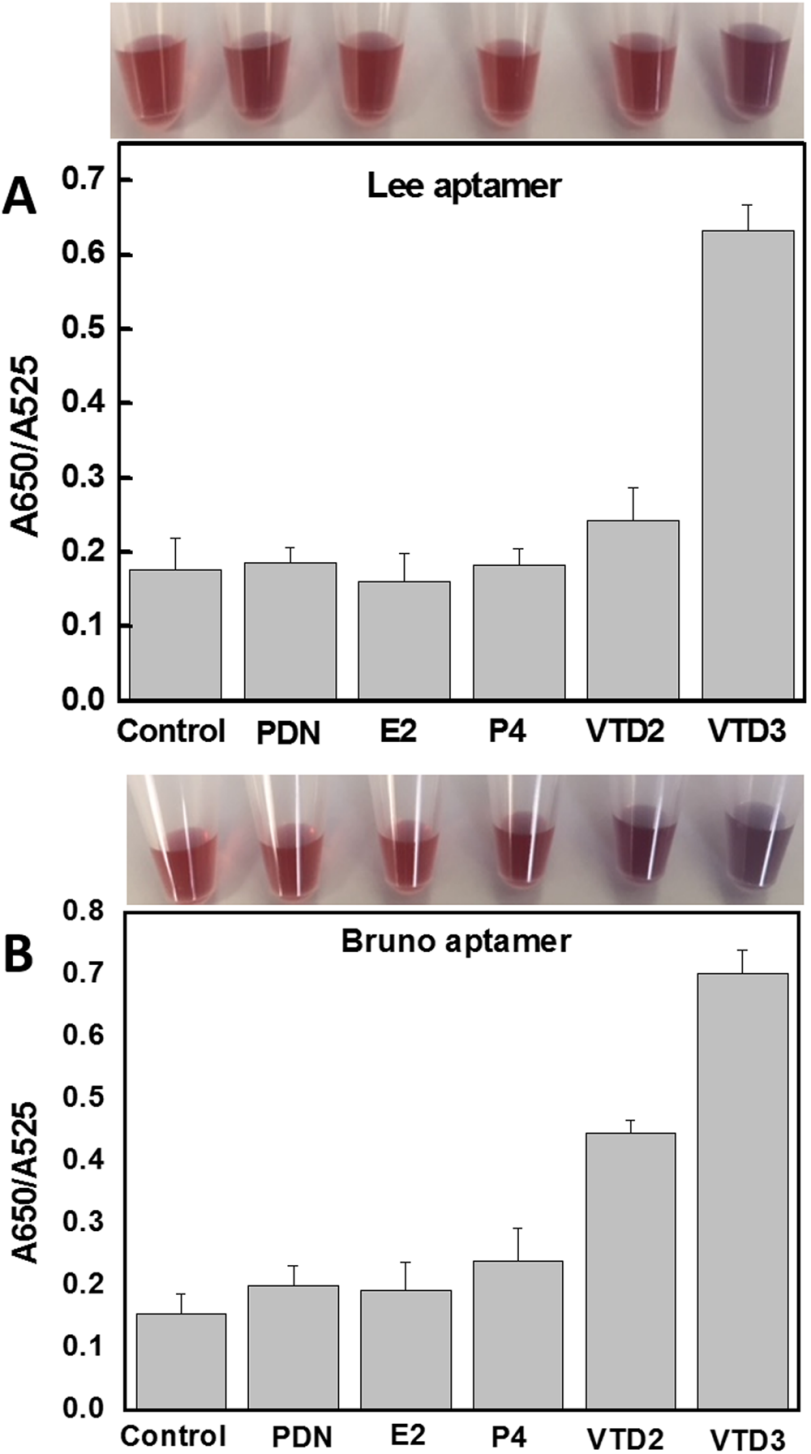
Selectivity examinations of interfering molecules at 600 nM and 10 µM using the AuNP-Lee aptamer and AuNP-Bruno aptamer, respectively. Error bars indicate standard deviation of the mean of three independent experiments starting from particle functionalization. Top panels show photos of the sensor responses to the interfering molecules. Note that the order of the different samples is the same order presented in the figure.

Having established sensitivity for VTD3, we examined the ability of both sensor systems to discriminate against structurally similar molecules likely to be encountered during the determination of the target compound in human blood. VTD2 is one of the major analogues and metabolites of VTD3 in the blood. It only varies by a methyl group and a double bond in the aliphatic part of the molecule. It is present at nM concentrations in the blood^43^. Progesterone (P4) and 17β-estradiol (E2) are natural reproductive hormones present at levels from high pM to low nM^59^. Prednisone (PND) is a steroid medication routinely prescribed to suppress the immune system for cancer patients and as an anti-inflammatory agent^60^. The molecular structures of these compounds are shown in the Supporting Information, Fig. S5. These compounds were individually incubated at 600 nM and 10 µM using the AuNP-Lee aptamer and AuNP-Bruno aptamer, respectively. These particular target concentrations were chosen because VTD3 triggered a strong colorimetric response when detected by the developed sensors. Figure 4A shows that the Lee aptamer is highly specific to VTD3, and only background signals are observed with the other interfering molecules. A more thorough examination of this sensor against VTD2 is shown in Fig. 2C, where no signals can be observed. The excellent selectivity found with this aptamer is broadly consistent with the study of Lee *et al*., which showed the lack of response when screening biologically relevant targets, although they did not examine the molecules examined in this study^37^. Figure 4B reveals that unlike the Lee aptamer-based sensor, the colorimetric sensor obtained with Bruno aptamer responded to VTD2 but not as strongly as to VTD3, and even weaker response was observed for P4. Only background response was observed for the other interfering agents. Our findings are broadly consistent with the results obtained by Bruno *et al*., in which the aptamer was found to recognize the various major analogues and metabolites of vitamin D3 without being able to distinguish between them^40^.

### Detection of VTD3 from human blood samples

**Figure 5:**
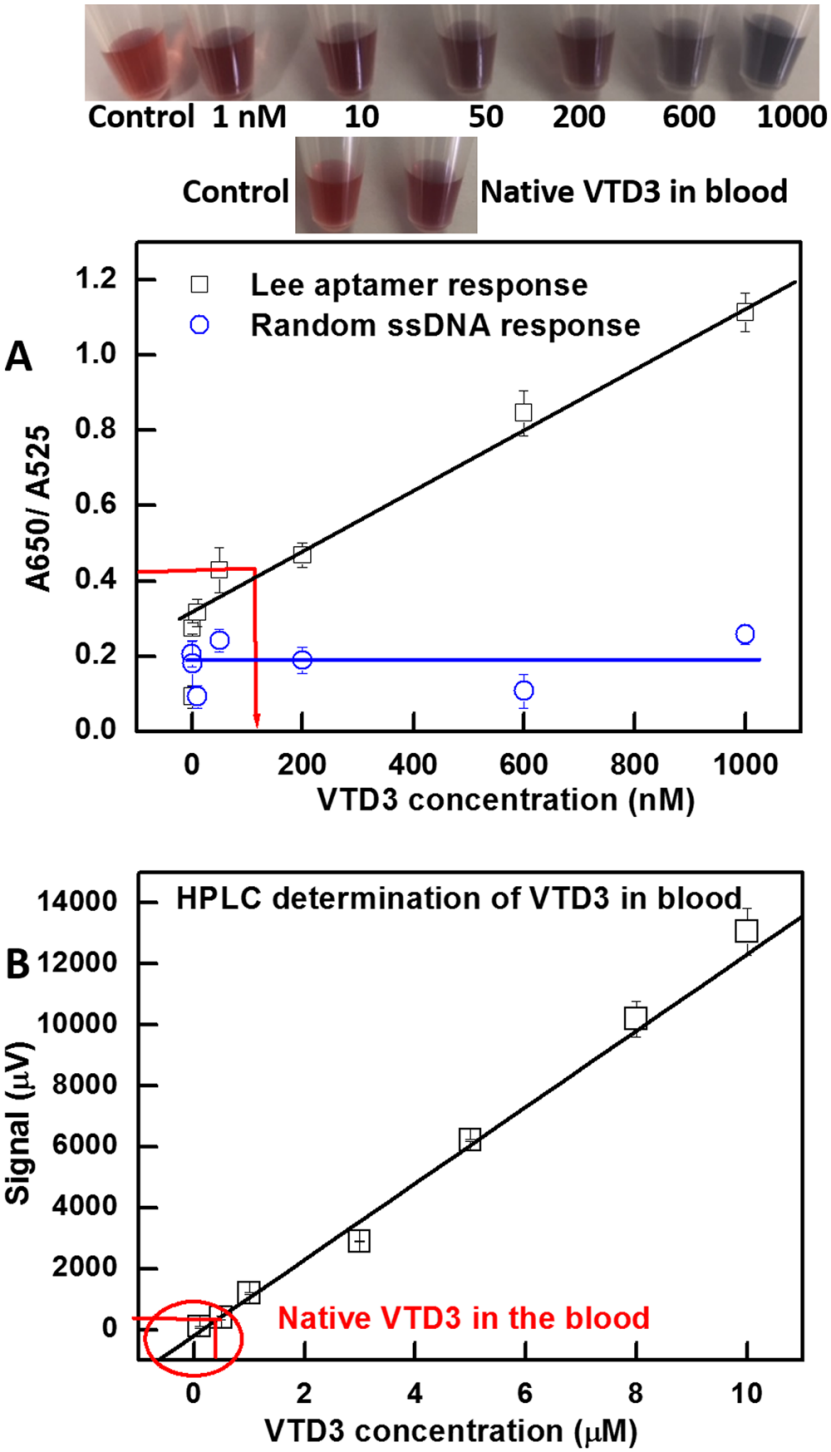
(**A)** Colorimetric aptasensor response with Lee aptamer towards a range of VTD3 concentrations spiked into and extracted from human blood using n-hexane, with redissolution in detection buffer under the centrifugation condition compared with control experiments using AuNP-70-mer random ssDNA exposed to the same experimental steps. Top panels show photos of the sensor response towards spiked VTD3 concentrations and its response to the native VTD3 concentration in the blood. Error bars indicate the standard deviation of the mean of three independent experiments starting from particle functionalization. (**B)** HPLC analysis of spiked VTD3 concentration in blood samples. Red arrows indicate the signal response for native VTD3 concentration extracted from blood. Raw UV-visible spectra and HPLC chromatograms are provided in the Supporting Information.

Finally, having demonstrated a strategy to enhance the sensitivity towards VTD3 with centrifugation and resuspension, we proceeded to detect VTD3 in the more challenging matrix of human blood using Lee aptamer, since it provides a low detection limit, more relevant analytical window, and better selectivity. To the best of our knowledge, a sensor with these remarkable properties has yet to be demonstrated to detect VTD3 from blood samples. Direct incubation of blood samples with AuNP-aptamer conjugate resulted in failure of the sensor due to the susceptibility of the AuNP surface to blood components, including proteins and cells that interfere with the detection scheme (Fig. S11). Instead, the desired hydrophobic VTD3 target and similar compounds were isolated from the rest of the blood matrix by solution-solution extraction using n-hexane, followed by redissolving the extract in the detection buffer (Fig. S7). The method is highly robust and achieves recoveries exceeding 97% (determined by HPLC of standard VTD3 concentration) in less than 10 min. Known VTD3 concentrations were spiked into and then extracted from a purified blood sample (free of VTD3). Figure 5A shows that the Lee system delivers the same detection limit, 1 nM, in samples extracted from human blood as those conducted directly in buffer solutions, Fig. 2C. Additionally, the sensor shows excellent linearity (R^2^ = 0.96) over the entire nM range and high reproducibility (error bars represent three independent experiments). Replacing the aptamer with a random 70-mer ssDNA sequence revealed that the signal arose from the aptamer-VTD3 interaction rather than nonspecific interactions. The developed calibration curve presented in Fig. 5A was used to directly determine the level of native VTD3 in a sample extracted from blood of a healthy donor. As seen, the resolved VTD3 concentration is estimated to be 115 nM (±10 nM), indicated by the red arrow in Fig. 5A. The resolved concentration is consistent with what was expected, as the normal VTD3 concentration in the blood ranges between 50 nM to 150 nM^45^. Colorimetrically comparing the response of the sample from native VTD3 with those of the calibration samples (top panel of Fig. 5A) would give a broad indication of the level of VTD3 in blood. Thus, this method could be used as a point-of-care application for rapid screening for VTD3 deficiency. Conducting spectroscopic measurements will help determine the precise quantification of VTD3 concentration circulating in the blood.

We confirmed our aptasensor-based determination of the native VTD3 concentration by conducting a standard HPLC analysis, routinely used in medical laboratories^45^. Figure S9 shows chromatograms of HPLC response to standard VTD3 concentrations and to the native VTD3 in blood. Note that the same extraction procedure described above was adopted for HPLC analysis. Figure 5B reveals that the concentration of the native VTD3 in blood is 120 nM (±22 nM). The resolved VTD3 concentration is above the detection limit of HPLC, experimentally determined for VTD3 (100 nM). The results of HPLC support our colorimetric determination of VTD3 in blood.

## Experimental Section

### Reagents and Chemicals

VTD3 and VTD2 were purchased from Carbosynth Limited, Berkshire - RG20 6NE – UK. Progesterone (P4), 17β-estradiol (E2), prednisone (PND), and chloroauric acid (HAuCl_4_) were purchased from Sigma-Aldrich. The first VTD3 aptamer in this study is named Bruno aptamer^40^. The sequence of this aptamer is a trade secret of Operational Technology Inc. and has the following inventory code: OTC Biotech Catalog number 070, VDA. The other VTD3 aptamer was selected by Lee *et al*.^37^ and named Lee aptamer in this study. This aptamer, along with the random ssDNA sequence used in the control experiments, was purchased from Alpha DNA. For the ssDNA preparations, the DNA samples were dissolved in deionized water (Milli-Q) and kept at −5 °C before use. Deionized water (Milli-Q) was used in all experiments (unless otherwise stated), and all other chemicals were of analytical grade. The sequences of the Lee aptamer and random ssDNA sequence are listed in the Supporting Information (Table S1). The secondary structures of the aptamers are presented in the Supporting Information Fig. S2.

### Methods

#### Synthesis of AuNPs

The particles were synthesized by the sodium citrate reduction reaction of HAuCl_4_^34,61^. In brief, 100 mL of 1 mM HAuCl_4_ aqueous solution was stirred vigorously at 250 °C, and a 10 mL of 38.8 mM sodium citrate solution was added immediately and left to react for 10 min. The solution was stirred for another 15 min at room temperature and then stored at 4 °C in the dark for other experiments. The concentration of the synthesized AuNPs was estimated to be 14 nM from the calculation based on the Beer−Lambert law using an extinction coefficient of 2.7 × 108 M^−1^ cm^−1^ at 525 nm^62^. The protocol results in AuNPs with a size of 13 nm in diameter and surface potential of −34 mV (transmission electron microscopy (TEM), dynamic light scattering (DLS), and phase analysis light scattering (PLS) data are shown in Fig. S1).

#### Aptamer adsorption to AuNPs

Prior to adsorption, the content of citrate ions adsorbed on AuNPs was minimized to render the stability of the system more sensitive to the presence of aptamers on the surface. This was done via a purification step where AuNPs were diluted by a factor of 1:10 using deionized water (Milli-Q), centrifuged at 13 000 rpm for 15 min (using Megafuge-40 centrifuge from Thermo Scientific; this centrifuge was used throughout the entire study), and then resuspended to the initial concentration in deionized water. Minimization of citrate ions was confirmed by PLS experiments where the ζ-potential value changed from −34 mV to −23 mV after purification. Three milliliters of the purified AuNPs was immediately mixed with 0.3 nmoles of the desired aptamer or ssDNA (taken from aqueous solution in Milli-Q water) to yield an aptamer concentration of 100 nM and an aptamer/particle ratio of 9:1, for a particle number of 2.5 × 10^13^. Note that a lower aptamer concentration affected the reproducibility of the sensor while the higher aptamer concentration resulted in a higher detection limit of VTD3. The NP-aptamer samples were prepared not more than 30 min prior to the sensing experiments, as longer particle to aptamer exposure time resulted in the failure of some sensing trials.

#### Optimization of salt concentration for sensing

The optimal salt concentration for sensing VTD3 was determined by conducting salt titration experiments for three different particle populations: bare AuNPs, AuNP-aptamer, and AuNP-aptamer + VTD3. One microliter of 0.5 M NaCl was progressively added to 100 μL samples. The samples were allowed to settle for 5 min, and UV-Visible absorption was measured, using the absorption ratio A650/A525 to evaluate the degree of aggregation. These experiments were also used to evaluate the adsorption and desorption of aptamer prior to and after target sensing.

#### Target detection

Stock solutions of VTD3 and interfering agents were prepared in pure ethanol before adding appropriate volumes to water and adjusting the overall ethanol content to 5%, to ensure sufficient target solubility (this solution is referred to as detection buffer). Aqueous target solutions were prepared fresh on a daily basis to avoid decomposition or precipitation of targets. Twenty microliters of the desired test concentration was added to 100 μL of AuNP-aptamer solution to obtain different VTD3 concentrations in a total reaction volume of 120 μL. Samples used as controls consisted of only water with 5% ethanol (blank buffer). Target binding was facilitated by incubating the samples for 10 min at room temperature. Then, the optimized NaCl concentration to trigger aggregation was added, followed by vortexing (for 5 min using Vortex Mixer MaxiMix™ from Thermo Scientific) or centrifugation (for 5 min at 13000 rpm) and resuspension. Finally, visual inspection after 10 min and measurement of UV-Visible absorption of 1 μL aliquots using a Thermo Scientific NanoDrop™ One/On Spectrophotometer were carried out.

#### Sensing VTD3 in human blood

Blood samples were collected from a healthy male, and VTD3 along with other hydrophobic molecules were isolated from the blood by extraction with n-hexane. Blood (0.5 mL) was mixed and shaken with an equivalent volume of n-hexane for 3 min, and the sample was centrifuged for 3 min at 13000 rpm (Fig. S7). The organic layer was removed and evaporated at 40 °C (3 min), and 0.5 mL of water with 5% ethanol was used to dissolve the dried sample. This portion of the original blood sample was used to determine the native VTD3 in blood. The efficacy of VTD3 extraction from blood by this simple method exceeded 97% (±1), confirmed by HPLC analysis of blood samples spiked with known concentrations of VTD3. The operation of the colorimetric sensor in blood (calibration curve) was tested by spiking blood samples (purified from native VTD3 by n-hexane extraction) with known VTD3 concentrations and extracting the target with the abovementioned n-hexane method. Control samples went through the same extraction procedure excluding the addition of VTD3. The remaining experimental steps were as described in the previous target detection section.

#### HPLC analysis

Reversed-phase HPLC Shimadzu chromatograph (10AD VP) with a UV-visible detector (SPD-10AV VP) equipped with the wavelength detection for VTD3 (275 nm) was used throughout the study. A C18 Raptor^TM^ analytical column (ARC-18 Catalogue No. 9314565, column length: 150 mm, inside diameter: 4.6 mm, particle size: 5 µm) was employed to separate the target molecules (VTD3 retention time, 0.7 min). The eluent (mobile phase) used was deionized water with 5% ethanol. The instrument flow rate and injection volume were 1 mL min^−1^ and 20 µL, respectively. Native blood concentration (after extraction with n-hexane and redissolving in deionized water with 5% ethanol) was determined by comparison with a calibration curve established from at least five different standard VTD3 concentrations. The standard concentrations underwent the same experimental protocol (extraction protocol) applied for the native VTD3 in blood to account for possible experimental variation. The detection limit of HPLC method was 0.1 µM (S/N = 3) determined experimentally under the same conditions adopted in this study. The efficacy of VTD3 extraction was tested and found to be not less than 97% (±1). This was confirmed by HPLC analysis of extracted blood samples spiked with known concentrations of VTD3. Note that there was no detectable VTD3 signal in the sample after the second n-hexane extraction from blood. This was an additional proof of the successful implementation of the extraction with n-hexane.

#### Transmission electron microscopy (TEM) and in solution surface potential (PLS) and size measurements (DLS)

TEM was performed on a JEOL 2010 microscope (200 kV). Five microliters of AuNP sample was cast onto a carbon-coated copper grid, followed by evaporation of the solvent under vacuum. Samples (120 μL) of various populations (bare AuNPs, AuNP-aptamer, and AuNP-aptamer + VTD3 (previously incubated at room temperature for at least 10 min) with no salt added were centrifuged at 13000 rpm for 5 min and resuspended in 1 mL of Milli-Q water. The samples were loaded into a folded capillary cell, which was inserted into a Zetasizer Nano ZS equipped with a 633 nm laser (Malvern Instruments, UK) and equilibrated at 25 °C for 2 min prior to surface potential measurements in triplicate (reported values are the average values with standard deviation not exceeding 1 mV). For size, 1 mL of each NP population sample was placed in a 1 cm path length disposable cuvette for size measurement.

## Conclusions

In this work, we report a novel and general method to eliminate the residual adhesion of nonbinding nucleotides of long aptamers to the surface of AuNP surface used in the aggregation colorimetric sensors for small molecules. Residual binding between the aptamer and AuNPs limits the practical applications of this useful and simple sensor by greatly diminishing its target sensitivity. By simply introducing a centrifugation and resuspension step after target detection, a remarkable enhancement of VTD3 colorimetric sensing was achieved for two previously isolated aptamers. Instead of a nonfunctional sensor, a sensor with a 1-nM level of detection, a wide dynamic range covering nM concentrations, robust operation, and high selectivity was achieved when implementing the centrifugation and resuspension method. The generality of this approach was examined for a different VTD3 aptamer and achieved at least 4-fold improvement (greater degree of aggregation response) compared to its original performance. The benefits of the proposed method became apparent by allowing accurate determination of VTD3 from human blood after n-hexane liquid-liquid extraction. HPLC results were consistent with those obtained from the colorimetric aptasensor. The work presented in this study provides a highly effective and selective point-of-care sensor for the nanomolar determination of VTD3 in blood. More broadly, the study provides an effective approach to enhance the performance of colorimetric AuNP aggregation sensors with no need for laborious sequence truncation and subsequent characterization steps.

## Electronic supplementary material

Supporting information
