# Supplementary material for: Colorimetric Aptasensor of Vitamin D3: A Novel Approach to Eliminate Residual Adhesion between Aptamers and Gold Nanoparticles
Source: Sci Rep. 2018 Aug 28;8:12947. doi: 10.1038/s41598-018-31221-y (PMC6113223; doi:10.1038/s41598-018-31221-y)
Supplement: Supplementary file 1 — Supporting information [file 41598_2018_31221_MOESM1_ESM.docx]

Supporting Information for:

**Colorimetric Aptasensor of Vitamin D3: A Novel Approach to Eliminate Residual Adhesion between Aptamers and Gold Nanoparticles.**

Omar A. Alsager^1*^, Khalid M. Alotaibi^2^, Abdullah M. Alswieleh^2^, and Baraa J. Alyamani^1^

^1^ National Center for Irradiation Technology, Nuclear Science Research Institute, King Abdulaziz City for Science and Technology, P.O. Box 6086, Riyadh 11442, Saudi Arabia

^2^ Department of Chemistry, College of Science, King Saud University, P.O. Box 2455, Riyadh 11451, Saudi Arabia

* Dr. Omar A. Alsager (corresponding author), E-mail: [oalsqar@kacst.edu.sa](mailto:oalsqar@kacst.edu.sa), National Center for Irradiation Technology, Nuclear Science Research Institute, King Abdulaziz City for Science and Technology, P.O. Box 6086, Riyadh 11442, Saudi Arabia.

**Figure S1. A)** TEM image showing the 13 nm prepared AuNPs. **B)** UV-visible absorption of AuNPs at 14 nM concentration (insert is a photo of AuNPs). **C)** DLS in-solution analysis of the sizes (18 nm) of the colloidal dispersion. D) PALS in-solution analysis of AuNP colloidal suspension surface potential (-34 mV).

**Figure S2. A)** shows the secondary structure of the Bruno aptamer (note that the secondary structure is adopted from Bruno et al. ^47^ (the actual composition of the ssDNA aptamer is a trade secret of Operational Technology Inc.) for the purpose of understanding the interaction between the AuNPs and the aptamer. **B)** Secondary structure of the Lee aptamer, determined using the web-based tool m-fold with the free-energy minimization algorithm. Circled fragments of the ssDNA show the unfolded part of the molecule likely to contribute to the residual adhesion identified in this study.

Table S1: Sequences used in this study

| **Name** | **Sequence** |
| --- | --- |
| Bruno aptamer | Trade secret of Operational Technology Inc. |
| Lee et al. 56-mer aptamer | 5ʹAGCAGCACAGAGGTCATGGGGGGTGTGACTTTGGTGTGCCTATGCGTGCTACGGAA-3ʹ |
| Random ssDNA (70-mer) | 5ʹAGGCCTAAGGGCATAATTAGCTCGAGCTCGAAAGGGGTTATATGATGATTTGAATTCATGGGGCCCGACT-3ʹ |

**Figure S3.** DLS (A and C) and PALS (B and C) characterizations of the sensor responses to VTD3 (at saturation condition) using AuNP-Lee aptamer (A and B) and AuNP-Bruno aptamer (C and D). E) A summary of the surface potential values of all samples examined in this study including bare AuNPs, AuNP-Lee aptamer, AuNP-Lee aptamer + VTD3 (1 µM) centrifuged and vortexed, AuNP-Bruno aptamer, AuNP-Bruno aptamer + VTD3 (20 µM) centrifuged and vortexed, AuNP-random ssDNA, and AuNP-random ssDNA + VTD3 (1 µM) centrifuged. **Figure S3 E** also shows additional control experiments where AuNP-aptamer was centrifuged while omitting VTD3 (referred to as no VTD3) to examine the dissociation of the aptamer sequences from the particles by centrifugation force only.

**Figure S4.** UV-visible spectra of the sensor performance using Bruno aptamer after independent incubation with increasing VTD3 concentrations and application of centrifugation and resuspension step.

**Figure S5.** Molecular structures of VTD3 and the interfering targets used to evaluate the specificity of the aptasensors developed in the study.

**Figure S6.** UV-visible spectra of the sensor response towards interfering molecules using Lee aptamer **(A)** and Bruno aptamer **(B)**. The centrifugation and resuspension step was used to examine the sensor specificity with both aptamers.

**Figure S7.** Photo representing the resulting layers of a blood sample after mixing and centrifugation steps with n-hexane.

**Figure S8.** UV-visible spectra of the sensor response (with Lee aptamer) to spiked VTD3 concentrations in human blood and native VTD3 concentration after extraction with n-hexane.

**Figure S9.** **A)** Raw HPLC chromatograms of the increasing concentrations of VTD3 spiked and extracted from human blood. **B)** Chromatogram of native VTD3 sample extracted from human blood.

**Figure S10.** Optimization of centrifugation time at a constant speed (13000 rpm). Centrifugation for 1.5 min and 3 min was too short and did not result in a complete isolation of the particles from the solution. However, centrifugation for 8 min and 12 min resulted in the irreversible formation of some aggregates. Thus, the optimized centrifugation time adopted in this study is 5 min.

**Figure S11.** Failure of the VTD3 aptasensor constructed with Lee aptamer to detect VTD3 directly in blood samples without extraction. Even 20 µM VTD3 concentration did not trigger any aggregation.

**Figure S12.** Salt tolerance experiments of bare AuNPs, AuNP-Random ssDNA (100 nM), and AuNP-Random ssDNA + VTD3 (1 µM) by vortexing and centrifugation sensing methods.

**Figure S13.** **A)** UV-visible spectra of the exposure of increasing concentrations of VTD3 to AuNPs under 25 mM salt concentration. **B)**  Comparison between the responses of Lee aptamer system (centrifuged-resuspension approach) with the interaction of VTD3 with bare AuNPs.

**Apparent dissociation constant of the aptamers investigated in this study**

As previously described in McKeague et al. ^59^, the KD, app values for the investigated aptamers and under the centrifuged based sensing and vortex based sensing were determined by obtaining saturation binding isotherms based on the titration of a constant aptamer concentration with an increasing concentration of VTD3 (the conditions used in the colorimetric sensor). The stoichiometry between VTD3 and aptamer was assumed to be 1:1 and the K, app was determined by applying the Langmuir model through nonlinear regression analysis by fitting the experimental data (using Origin 6.0) with the following equation:

$\boldsymbol{Y=}\boldsymbol{B}_{\boldsymbol{max}} \frac{\boldsymbol{X}}{\boldsymbol{X}\boldsymbol{+}\boldsymbol{K}_{\boldsymbol{d, app}}}$ **(1)**

where X is the concentration of VTD3, Y is the signals generated from the sensor (A650/525), and B_max_ is the maximum signal of the absorption ration A650/525.

It was found that there was no resolvable binding between lee aptamer and VTD3 when implementing the vortexing method. However, a Kd, app value of 220 nM was calculated from the non-linear fit of the data representing the centrifugation and resuspension approach, **Figure S14A**. Similarly, a better Kd, app was calculated for Bruno aptamer when the centrifugation and resuspension was used compared to the vortexed based sensing (5 µM vs. 9.2 µM respectively), **Figure S14B**. These results are consistent with our previously noted observation with the overall sensor performance and sensor sensitivity. The centrifugation and resuspension approach seems to eliminate the residual adhesion of non-binding aptamer segments to AuNPs, which makes the sensor more sensitive to the VTD3 concentrations.

**Figure S14.** Calculation of apparent dissociation constant (Kd, app) when implementing **Equation 1** and non-linear regression using Origin software for **A)** Lee aptamer and **B)** Bruno aptamer with the two sensing methods centrifugation and vortexing. Error bars represent standard deviation of the mean of three independent experiments.
